# Phase Change Metasurfaces by Continuous or Quasi-Continuous Atoms for Active Optoelectronic Integration

**DOI:** 10.3390/ma14051272

**Published:** 2021-03-07

**Authors:** Zhihua Fan, Qinling Deng, Xiaoyu Ma, Shaolin Zhou

**Affiliations:** 1 Chengdu Research Institute, Sichuan University of Arts and Science, No. 519 Tashi Road, Dazhou 635000, China; fanzh@sasu.edu.cn (Z.F.); ccoe@rccoe.com (X.M.); 2 School of Microelectronics, South China University of Technology, No. 381 Wushan Road, Guangzhou 510640, China; eeqldeng@mail.scut.edu.cn; 3 Chongqing Co-Core Optics & Electronics Technology Institute Co., Ltd., Panxi Road, Chongqing 400021, China

**Keywords:** optoelectronic integration, active photonics, dynamic wave control, continuous and quasi-continuous metasurfaces

## Abstract

In recent decades, metasurfaces have emerged as an exotic and appealing group of nanophotonic devices for versatile wave regulation with deep subwavelength thickness facilitating compact integration. However, the ability to dynamically control the wave–matter interaction with external stimulus is highly desirable especially in such scenarios as integrated photonics and optoelectronics, since their performance in amplitude and phase control settle down once manufactured. Currently, available routes to construct active photonic devices include micro-electromechanical system (MEMS), semiconductors, liquid crystal, and phase change materials (PCMs)-integrated hybrid devices, etc. For the sake of compact integration and good compatibility with the mainstream complementary metal oxide semiconductor (CMOS) process for nanofabrication and device integration, the PCMs-based scheme stands out as a viable and promising candidate. Therefore, this review focuses on recent progresses on phase change metasurfaces with dynamic wave control (amplitude and phase or wavefront), and especially outlines those with continuous or quasi-continuous atoms in favor of optoelectronic integration.

## 1. Introduction

To date, versatile electromagnetic (EM) wave control remains an almost everlasting topic for applications ranging from the visible to the microwave region. In recent decades, metasurfaces such as the planar or two-dimensional (2D) version of metamaterials with deep subwavelength thickness, have proven to be effective and promising in wave regulation in an almost arbitrary way, similar to their traditional volumetric counterpart, but hold the special ultra-thin nature that facilitates the compact integration of devices with hybrid architectures. Instead of tuning the propagation phase accumulated through a bulk region of material as the conventional photonic devices, metasurfaces aim at tailoring the abrupt change of amplitude, phase and polarization locally and in an accurate pixel-wise manner, via various subwavelength meta-atoms that are periodically or randomly distributed, e.g., nanorods, nanogratings, nano-trapezoids, catenary resonators, LC circuit resonators, split ring resonators, ring resonators, cross resonators etc.

Especially when competing with nanoelectronic technologies for increasingly high-speed data processing, communication and interconnection, etc., compact optoelectronic device integrations or even all-optical integrations become more desirable due to the ultra-high speed and large bandwidth. In this situation, photonic metasurfaces show more prospects and outperform their volumetric versions mainly due to the ultra-thin and easy-to-integrate characteristics. Therefore, a plethora of meta-devices are demonstrated for both amplitude [1] and phase regulation [2], from the visible to terahertz (THz) and microwave range, such as ideal frequency selection [3,4,5], perfect absorption [6,7,8,9], beam steering or deflection [10,11], flat lensing [12,13], vortex beam generation [14,15,16], optical activity [17,18], holography [19,20,21], 3D color holography [22,23] and nonlinear effect [24,25] etc.

However, for applications of optoelectronic or all-photonic integrations, dynamic functionalities are imperative since the optical responses usually need to be actively switched, modulated or flexibly tuned by external stimuli. Therefore, active metasurfaces with diverse reconfigurable functionalities were extensively explored in recent years, such as tunable filtration or absorption [26,27], beam steering [28,29], switchable lensing [30] and switchable photonic crystals [31]. In principle, all those actively reconfigurable metasurfaces can be constructed by: (i) embedding active materials or components into hybrid device architectures, or (ii) directly structuring into thin films of active materials (e.g., graphene [32,33,34], phase change chalcogenides [35,36,37]). After a close inspection and classification, such active materials or mechanisms include the liquid crystal (LC) [38], MEMS [39,40], semiconductors [41,42], the 2D materials family represented by graphene [43,44,45], atomic-thin-layer direct tuning of 2D electron gas [46], conductive metal oxide (i.e., Indium Tin Oxide ITO) [10,47,48,49], magnetic or ferromagnetic materials [50,51], varactor arrays [52,53,54], and phase change materials (PCMs) [28,29,30,36,55,56,57,58]. Among these mainstream options, the LC-based methods are commonly used for conventional optical modulation, but with intrinsic obstacles in CMOS-compatibility and high-speed operations, especially for integrated photonics. The MEMS-integrated metadevices exhibit a large modulation depth with great flexibility and low power consumption, but usually with high complexity in design and nanofabrication. The schemes using semiconductors or 2D materials depend on relatively large bias voltage due to low modulation depth. As a result, PCMs-based approaches turn out to be a prominent and practical category for dynamic tuning that had been the most intensively explored in a wide range of applications, due to the overall trade-offs among modulation depth, power consumption, operation speed, CMOS-compatibility and complexity etc., as well as the flexible tunability of PCM in both electrical and optical properties. Furthermore, similar schemes were also employed for active control in silicon photonics by integrating active materials (e.g., graphene [59,60,61] or PCM [62,63,64,65]) into integrated photonic devices. Due to their prospects in micro and nanophotonics, a few review papers were recently reported on both on-chip based active photonic devices [65,66,67,68,69] and tunable metasurfaces [48,70,71,72,73,74,75,76,77,78,79].

In the family of PCMs, vanadium dioxide (VO_2_) somehow grabs earlier attention for active photonic or EM devices due to its lower temperature for easier and reversible insulator–metal phase transition [58,80,81,82,83,84]. However, due to the volatile nature of VO_2_, which means its metallic or insulative state cannot be maintained without external excitations, the unique traits that lie in the non-volatile chalcogenide PCMs are more desirable, especially for integrated optoelectronics. Other prevailing performances in chalcogenide PCMs include long-term stability in both amorphous and crystalline states, ultrafast phase transition at the nanosecond level, a large number of phase changes in millions of repeatable cycles, and CMOS compatibility [85]. In the early days, chalcogenide PCMs have been pursued for applications in electronic storage and optical memory, such as the phase change memory [85,86] and the optical compact disks (CD) and digital versatile disks (DVD) [87]. Upon a phase transition of chalcogenide PCMs, the germanium antimony telluride (GST) alloy with varied ratios of compositions [88] typically assumes large contrasts in both optical and electrical properties (i.e., refractive index, permittivity, conductivity etc.), which specially facilitate the active optoelectronic or even all-photonic integration. As a result, the merits of PCMs integrated metasurfaces and devices enable versatile optoelectronic integration for dynamically reconfigurable wave–matter interactions, i.e., active amplitude, phase (or wavefront) and polarization control.

Therein, one minor issue is the relatively large optical losses co-existing with a large refractive index contrast for typical chalcogenides (e.g., G_2_S_2_T_5_). Thus, more candidates from the chalcogenide family emerge with lower optical loss and prove valid to improve the performance for visible-range applicability [89,90,91]. The second issue lies in most traditional metasurfaces constructed by discontinuous meta-atoms, which inevitably introduce low efficiency, the involved amplitude interval and phase noise, due to discrete wavefront sampling by discontinuous meta-resonators even at the subwavelength level. So metasurfaces with continuous or quasi-continuous atoms are preferred for practical use due to higher efficiency as well as the conveniences for optoelectronic integration. The third concern is the mutual interactions or couplings between photonic and electrical elements in the optoelectronic integrated architecture of metasurfaces. The ideal case is that photonic elements of meta-atoms and electrical elements of metal electrodes function independently without cancelling out or influencing each other. In certain scenarios, the continuous meta-atoms simultaneously act as electrodes that can be connected externally in arrays to facilitate pixel-wise addressing and electrical control, e.g., spatial light modulation, displayer and so on.

Therefore, this review aims at PCMs-integrated metasurfaces for active amplitude and phase tuning in terms of device architecture and functionality, and specifically features recent advances in continuous or quasi-continuous metasurfaces facilitating the strategies of optoelectronic integration. The PCM origins with unique tunable properties for photonic devices are discussed first in the next section. Then, recent advances in diverse PCM-integrated metasurfaces for dynamic amplitude and wavefront controls are reviewed, respectively. Specifically, in the section that follows, a few strategies using metasurfaces with continuous or quasi-continuous meta-atoms for optoelectronic integration are outlined. Finally, the conclusion section outlooks several possible trends in the near future.

## 2. Origin of PCM for Active Photonics

To start with, vanadium dioxide (VO_2_) might be the first group of PCMs that attracts intensive interests to construct active photonic devices for a long while [82,92,93,94,95]. Upon a reversible metal-to-insulator phase transition (MIPT) that can be triggered by an electric field, optical or thermal heating, VO_2_ exhibits a large contrast of electrical conductivity and optical constant [96]. Such a unique phase transition process that occurs near room temperature (~68°) was fundamentally studied in a few literatures [97,98].

Due to the distinct contrast of electrical conductivity, the insulative VO_2_ (before phase transition) shows ultra-high transmission beyond the infrared (IR) range, but the metallic VO_2_ (after phase transition) change significantly to become highly absorptive in almost the whole spectral range. Therefore, a diversity of VO_2_-hybrid active metamaterials or metadevices emerged from the visible to THz and microwave range, e.g., tunable absorbers [83], THz modulators [81,99], THz switches [94], wavefront engineering [95] etc. In addition, a few review papers about VO_2_-hybrid active metamaterials had been reported [56,100,101,102]. However, due to the volatile nature of VO_2_, its metallic phase vanishes as long as the external stimuli are withdrawn.

Therefore, the chalcogenide glass PCMs, typically the germanium (Ge) antimony (Sb) telluride (Te) alloy (GST), dominates more scenarios due to its non-volatile nature, i.e., the amorphous or crystalline state still holds in absence of external stimuli, leading to almost zero static power-consumption. Upon a typical phase transition, GST similarly exhibits large electrical and optical contrast between the amorphous and crystalline states, in both the real and imaginary parts of refractive index or dielectric constant. Additionally, an appreciable loss can be found from the visible to the near-IR range, facilitating certain applications where absorption is desired. Overall, those superior performances enable a wide branch of electronic and photonic devices with pronounced tuning ability and increased freedoms for versatile manipulations [85,103,104]. For applications with varied pursuits in optical or electrical properties, a compromise among several considerations or priorities has be to be reached for GSTs with varied proportions of ternary composition, i.e., rapid phase transition (especially the crystallization), long-term chemical and thermal stability, a large number of reversible cycles of phase change, large contrast (real part) with relatively low or high loss (imaginary part) etc.

**Figure 1:**
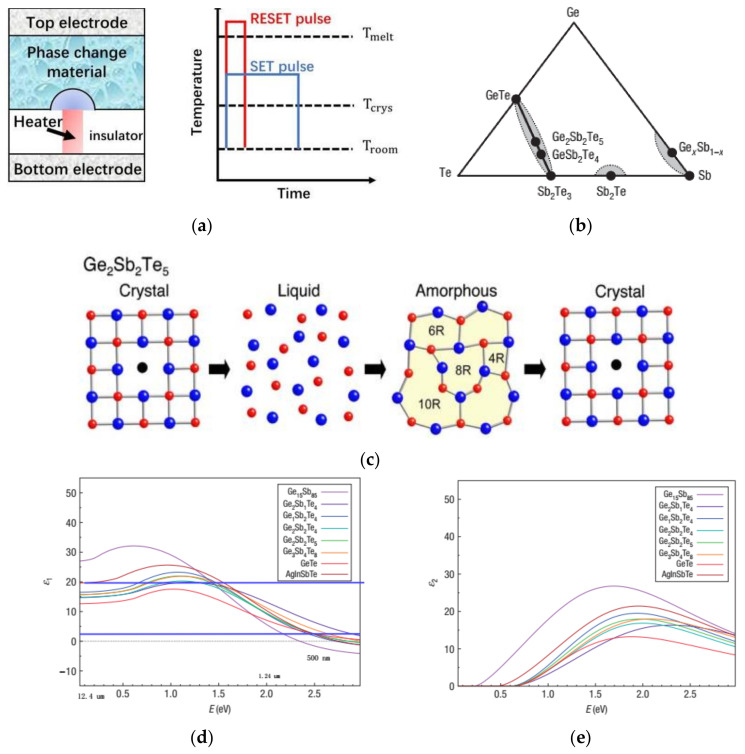
The schematic of phase transition in chalcogenide phase change materials (PCMs); (**a**) a typical cell of phase change memory and the curve of phase transitions triggered by electrical pulses, reproduced with permission from; (**b**) the phases diagram of germanium (Ge) antimony (Sb) telluride (Te) alloy (GST) with varied ternary compositions. The often-used GST can be found along the pseudobinary route between GT and S_2_T_3_, reproduced with permission from [88]; (**c**) an artistic impression of typical phase transition process of G_2_S_2_T_5_, reproduced with permission from [105]; (**d**,**e**) the optical constants of commonly used GSTs, both reproduced with permission from [106].

As a special group of PCMs, the GST family attracted intensive interests from electronics to photonics. In recent decades, early GSTs with slow speed of crystallization to match the speed of CD writer have been dominantly used for optical disk/storage [87,88]. Subsequently, an increased speed of crystallization makes the GST alloy a good candidate for the next generation of non-volatile electronic memories, i.e., phase change random access memory (PCRAM) [85,86], which rival the mainstream flash and dynamic random-access memory (DRAM). In principle, the amorphous-to-crystalline transition is triggered by a long (electrical or laser) pulse with lower amplitude to heat GST above its crystallization temperature *T_crys_* (or glass temperature *T_g_* elsewhere), termed as “SET” for a low-resistance state in PCRAM, as shown in Figure 1a. For the reserve process of crystalline-to-amorphous phase transition, a short pulse with higher amplitude is needed to heat GST above its melting temperature *T_melt_*, termed as “RESET” for a high-resistance state in PCRAM.

To date, as intensively studied by Wuttig and Taubner’s group [88,104,106,107], a few representative GST alloys, i.e., the G_1_S_4_T_7_, G_2_S_2_T_5_ and G_3_S_2_T_6_ along the pseudobinary route between GT and S_2_T_3_ in Figure 1b, have been the most favorably used for active photonic devices. As revealed by early reports [108,109], GSTs with varied ternary compositions exhibit distinctly different dynamic performances and optical properties. For a brief summary, for GST with a higher ratio of Sb along the pseudobinary route in Figure 1b, the crystallization speed increases, the temperatures of *T_crys_* and *T_melt_* decrease and the amorphous state becomes less stable. For a good compromise, G_2_S_2_T_5_ shows a fast speed of crystallization (~tens of ns) and moderate high *T_crys_* (~150°) to ensure a long-term stable amorphous state [88]. In this situation, a variety of GSTs with slightly different compositions were employed for photonic devices, and their optical constants had been well characterized [106], as shown in Figure 1d.

## 3. Active Amplitude Control

### 3.1. Tunable Transmission/Reflection

The pioneering work based on chalcogenide-PCM was reported by Zheludev’s group in the construction of an electro–optic metasurface switch by integrating the asymmetric split ring resonators (SRR) with gallium lanthanum sulfide (GLS) [110]. The near-IR transmission and reflection spectrum of Fano resonance can be electrically tuned (~10 ms, >45 V) with a contrast ratio of 4:1 and obvious frequency shifts can be observed during the GLS amorphous-to-crystalline phase transition. Subsequently, they improved the SRR device setup for a concurrent probe and control by using high-intensity laser pulses instead of electrical control and replacing the chalcogenide film of GLS with G_2_S_2_T_5_ for an all-optical meta-switch with bidirectional reversible control [63], shown in Figure 1a. It is worth noticing that, for both schemes, the optical and electrical pulses need to be precisely optimized with varied durations and intensities for effective and rapid phase transition between the amorphous and crystalline states.

**Figure 2:**
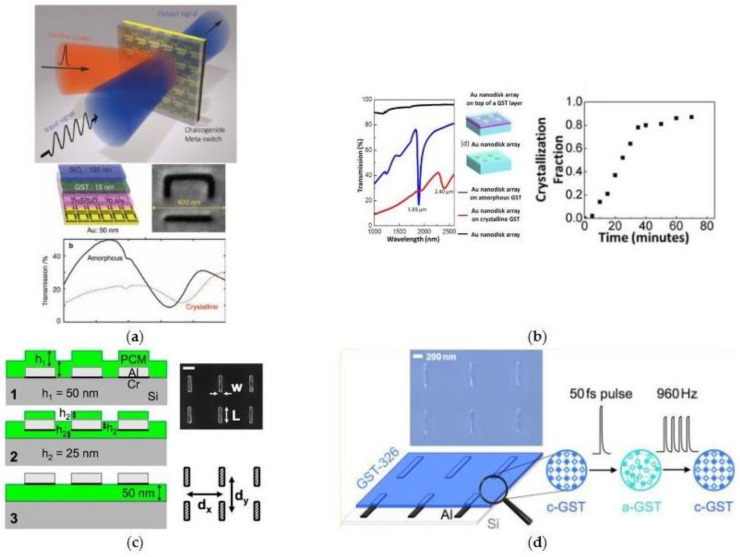
Early reported work on active metasurfaces with tunable amplitude transmission. (**a**) A near-IR all-optical meta-switch by integrating split ring resonators (SRR) meta-atoms with G_2_S_2_T_5_; (**b**) the GST—Au hybrid plasmonic crystal with continuous transmission tuning by corresponding fractions of crystallization—(**a**,**b**) are reproduced with permissions from [63,112], respectively; (**c**,**d**) switchable mid-IR antenna resonance using G_3_S_2_T_6_, reproduced with permission from [55,112].

Meanwhile, by controlling the baking time in a thermal-stimulated phase transition process, M. H. Hong’s group demonstrated a G_2_S_2_T_5_ hybrid metasurface working at intermediate states with varied fraction of crystallization between the amorphous and crystalline states [111]. By a close inspection of tunable resonance/transmission peak of the G_2_S_2_T_5_-hybrid plasmonic crystal, the relationship between the fraction of crystallization and baking time is quantitatively explored, as shown in Figure 2b. In addition, another type phase change chalcogenide in the PCM family, G_3_S_2_T_6_, with lower mid-IR loss, was also introduced into the active metasurfaces based on the plasmonic resonances of Al antenna array [56,112], shown in Figure 2c,d. A femtosecond laser pulse (800 nm, 50 fs and repetition rate of 960 Hz) was also used to trigger the reversible amorphous–crystalline phase transition for optically tunable transmission [112].

Other than the frequently used nano-antennas, SRRs or crosses for a GST-integrated metasurface hybrid framework, other atoms such as nano-holes, squares and rings were also employed for active tuning, e.g., the tunable extraordinary transmission (EOT) of visible and near-IR light by both electrically and optically induced GST phase transition [113,114], the mid-IR transmissive filter [115] and the lately reported mid-wave spectral filter [116], etc. In addition, due to the appreciable visible and UV loss of GST, most GST-based devices were demonstrated in the middle-IR range except a few that covered UV, visible and near-IR range [117,118,119].

### 3.2. Tunable Absorption

As a special subcategory for tunable transmission or reflection, tunable perfect absorption is always realized in a well-known metal–insulator–metal (MIM) architecture by the construction of a PCM hybrid metasurface. In such a configuration, a reflective spectrum was minimized because the transmission of the MIM meta-device was truncated by the metal ground layer. Following Landy’s pioneering proposal in 2007 [120], MIM absorbers were intensively constructed by meta-atoms with varied geometries for plasmonic enhanced sensing [121,122], photodetection [123] etc. Furthermore, to conquer the narrow band nature of early MIM absorber, broadband absorption was realized by the hybridized design of multiple meta-atoms [124]. To name a few, exquisite schemes by Luo’s group and others were used to construct the extremely broadband perfect absorption, e.g., dispersion engineering [1,125,126], the impedance matching [127] and diffraction/interference engineering [7,8,128] etc.

**Figure 3:**
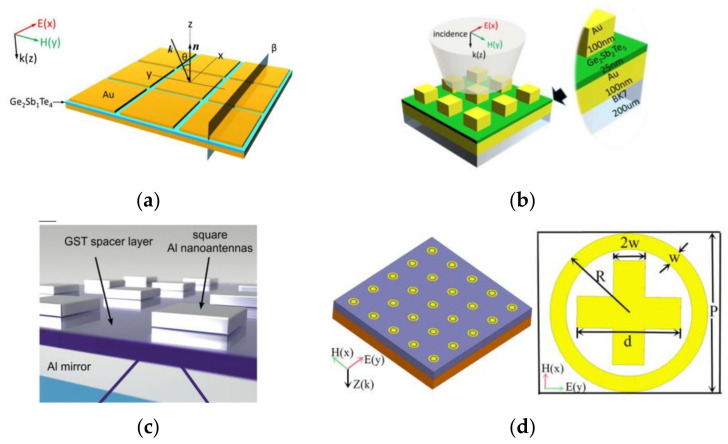
The PCM hybrid metal-insulator-metal (MIM) tunable absorbers by different meta-atoms or PCMs. Au squares on top of (**a**) G_2_S_1_T_4_ and (**b**) G_2_S_2_T_5_ spacer with metal ground underneath; (**c**) Al antennas on top of G_3_S_2_T_6_ spacer layer for mid-IR MIM tunable absorber; (**d**) a THz tunable absorber for the metaswitch using composite meta-atoms—(**a**–**d**) are reproduced with permission from references [130] (@The Optical Society), [27,129,131] respectively.

Towards this trend, the PCM hybrid tunable absorber was proposed by replacing the regular insulative layer with GST film in the MIM configuration. G_2_S_2_T_5_ and G_2_S_1_T_4_ were used in a few pioneering demonstrations of MIM tunable perfect absorber by Cao et al. [26,129,130], as shown in Figure 3a,b. Upon an amorphous-to-crystalline phase transition, the absorption peak by localized magnetic and electric dipole resonances shifted distinctly, due to a considerable change in the permittivity or optical index of the GST layer. Later, Giessen’s group demonstrated another mid-IR tunable perfect absorber using Al antennas and GST-326 as the spacer [131], shown in Figure 3c. For applications in the THz range, one type of exquisite composite meta-atom composed of resonant crosses and rings was also used to demonstrate the meta-switch by Zhou et al. [27,132].

### 3.3. Tunable Thermal Radiations

It is worth noting that the PCM–MIM hybrid meta-devices can be also used for tunable thermal emission, because the absorptivity of materials directly determines their emissivity and perfect absorbers simultaneously act as quality emitters or radiators. For example, Qu and Du et al. presented a type of mid-IR tunable metasurface for dynamic thermal emission control [133,134,135] or thermal camouflage [136]. By changing the heating time for different levels of crystallizations, intermediate states of GST can be obtained, and the emissive peak and emissivity can be quasi-continuously tuned [133]. Overall, such meta-devices may provide an alternate approach for nanophotonic engineering the far-field thermal emission, which has been a ubiquitous and fundamental process for energy harvesting and radiative cooling [137].

### 3.4. Tunable Circular Dichroism

As a special subcategory, chiral metamaterials or metadevices act as appealing platforms for handedness control in chiral sensing, polarization engineering and optical activity etc. [138]. Therefore, recent attempts also aimed at flexibly reconfigurable circular dichroism (CD) by resorting to PCMs–metamolecules hybrid active chiral devices in mid-IR and THz regimes.

In the mid-IR range, Yin et al. demonstrated active chiral behavior by using a Born–Kuhn type of chiral plasmonic dimmer together with the GST-326 layer sandwiched in-between two vertically displaced, corner-stacked and orthogonal arranged gold nanorods [139]. Different transmittances and reverse CD degrees were demonstrated for the left- and right-handed circular polarization (LCP or RCP) at either amorphous or crystalline state. Upon a phase transition of GST film, both transmittance and CD spectra underwent a large redshift. In the THz range, Wang et al. recently demonstrated the actively controllable optical activity using a VO_2_-grounded MIM setup for THz waves [140]. Distinct phenomena of tunable CD or transmission spectra can be observed upon the metal–insulator phase transition of VO_2_.

### 3.5. Pixelated Dynamic Tuning for Color Display

Currently, most devices for active amplitude control, as discussed above, are global based, i.e., phase transition occurs over the whole device non-selectively. However, in some cases, pixel-by-pixel local tuning of each meta-unit or subsection is imperative for spatially variant wave control/modulation, e.g., color display. In this regard, there is a pursuit to pixel-wisely address individual atoms or resonators for programmable or even smartly controllable metasurfaces.

**Figure 4:**
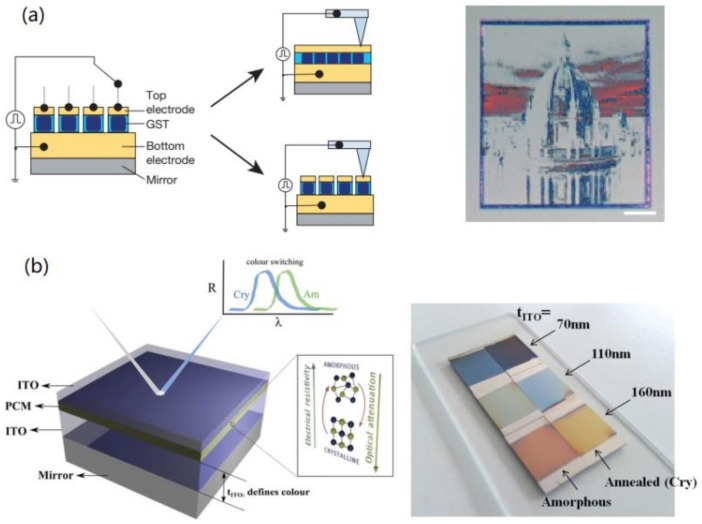
The pixel-wisely programmable GST/dielectric metasurface for color display: (**a**,**b**) reproduced with permissions from [141,142].

In 2014, the pioneering work by Hosseini et al. demonstrated a hybrid metasurface of ITO/GST/ITO framework for greyscale and color imaging [141], as shown in Figure 4a. The F–P cavity based meta-atoms with GST embedded were selectively addressed and electrically switched by the conductive tip of atomic force microscopy (AFM). As a result, a dielectric reflective display film with greyscale and color image was patterned by driving the AFM tip in a programmable manner. Furthermore, they tended to improve the depth modulation and resolution in an off-line color modulation mode, by replacing GST-225 with one type of growth-dominated phase-change alloy, Ag_3_In_4_Sb_76_Te_17_ (AIST) [142]. A similar setup with F–P cavities was used for better performances, i.e., non-binary color rendering, resolutions to 300 nm in scanning mode and <50 nm in pixel-by-pixel mode. Etc.

Other than electrical control above, most works in the following years resort to optical heating, e.g., the spatially controllable femtosecond laser scans [143,144], to realize the pixelated switching or programming of individual atoms. Also differing from the dielectric/PCMs F–P setup above, the GST-resonators hybrid architectures were used to construct the spatially programmable meta-atoms. As typical examples, Taubner and Wright’s group demonstrated a series of programmable metasurfaces for color display or spectral imaging using diverse schemes of hybrid meta-atoms, including the PCM hybrid nanoantennas [145], the MIM setup [146], and PCMs-embedded dielectric nanodisks [147] etc. Very recently, Ann-Katrin et al. proposed a scheme for the localized phase transition of germanium telluride (GT) by using the thermal scanning probe [124]. In numerical calculation, partial crystallization by scanning-probed-induced localized phase switching enabled a broadband tunable reflectance or absorption.

## 4. Active Wavefront/Phase Control

Differing from the previous discussion about active amplitude regulation, dynamic wavefront control resorts to actively shaping the phase distribution/gradient by tuning the Pancharatnam–Berry (P–B) or geometric phase, propagation phase or both. By active phase control, a diversity of typical wavefront-tunable devices were demonstrated, such as beam steering [29,148], switchable lensing/focusing [30,149], tunable spin angular momentum (SAM) and orbit angular momentum (OAM) coupling [76,150], tunable optical activity or vortex beam [151], and switchable holography [150]. A few of those typical categories are briefly summarized in this section, with a special focus on those based on the most commonly used GSTs, the GST alloy.

### 4.1. Tunable/Switchable Steering

In principle, beam steering can be achieved by actively redistributing the linear phase profile of one state to another with a varied constant phase gradient. Inspired by Huang and Chen’s work [152], the P–B phase determined that light deflection or propagation can be controlled by periodically arranging a series of spatially rotated nanorods to produce a linear phase profile. Therefore, the beam steering devices can be configured by integrating active materials into the typical P–B phase architecture.

**Figure 5:**
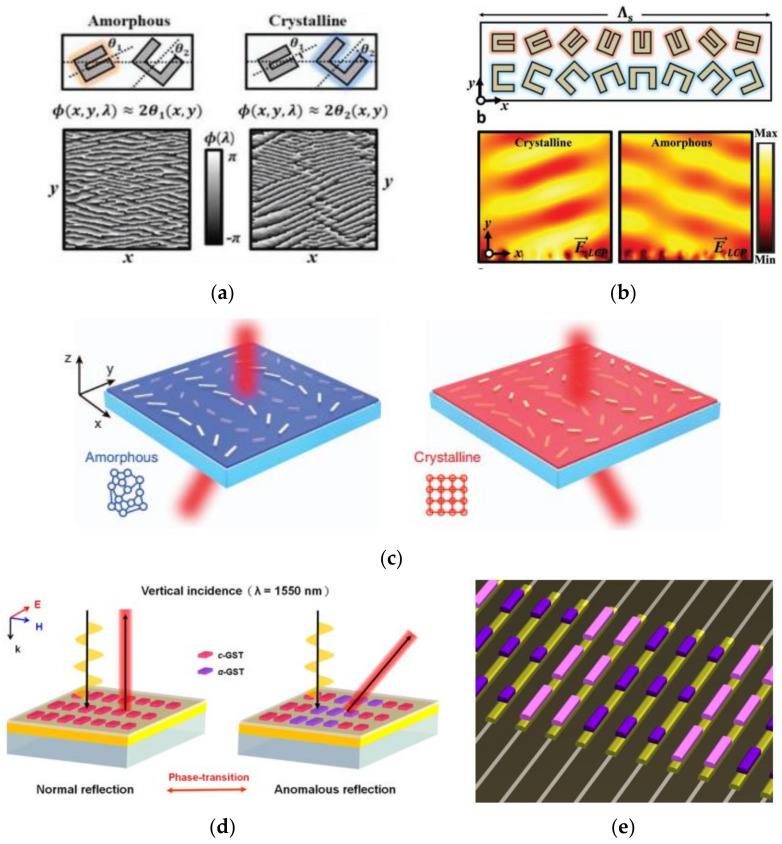
Beam steering by GST hybrid devices with tunable phase modulation. (**a**) The U-shaped G_2_S_2_T_5_ nanoantennas with different sizes and orientations (**b**) are arranged adjacently, and dominate alternately for opposite deflection in the amorphous or crystalline state; (**a**,**b**) reproduced with permissions from [148]; (**c**) two neighboring antennas A and B interact with the incident light of 3.1 μm alternately in the amorphous and crystalline states, reproduced with permission from [149]; (**d**) all-dielectric G_2_S_2_T_5_ metasurface with switchable steering at 1.55 μm; and (**e**) selective access and modification of local antenna by electrical pulse via conductive wires—(**d**,**e**) reproduced with permissions from [28].

In this trend, Choi et al. proposed a near-IR broadband wavefront switch by using two sets of U-shaped G_2_S_2_T_5_ nano-antennas with different sizes on quartz substrate [148]. As shown in Figure 5a, two sets of U-shaped antennas were multiplexed with a different orientation angle θ_1_ andθ_2_. Two sets of antennas dominate alternately in the amorphous or crystalline state with maximized cross-polarization (CPT), giving rise to a phase profile of θ_1_(x, y) or θ_2_(x, y), respectively. Namely, antenna 1 dominates with large CPT but antenna 2 show almost zero CPT in amorphous state, and vice versa. When two neighboring antenna were arranged with reverse orientations, shown in Figure 5b, phase transition between amorphous and crystalline states obviously induced opposite beam deflection. Subsequently, Yin et al. proposed another G_3_S_2_T_6_ hybrid plasmonic metasurfaces for mid-IR beam steering by using two sets of linear nanoantenna [149]. In a similar manner, antenna A and B with different lengths were arranged adjacently with opposite orientations. Each of them resonated with the incident light of 3.1 μm alternately in the amorphous and crystalline states and deviated left and right, respectively, as shown in Figure 5c.

According to the generalized Snell’s law [153], anomalous deflection (reflection or refraction) can be obtained by tailoring the abrupt phase profile in whatever manner, e.g., the P–B phase, the propagation phase, resonant phase or the hybrid mode. Instead of using the P–B phase, Tsai’s group presented on types of all-dielectric GST-hybrid phase change metasurface with switchable phase control for beam steering in a different manner [28]. Shown in Figure 5d, the G_2_S_2_T_5_/dielectric nanorods were used to replace the metal atoms in conventional MIM setup, i.e., GST atoms and metal ground sandwiched by a spacer layer. Upon a crystalline-to-amorphous phase transition, the normally reflected beam was switched/steered towards the anomalous angle of −40°, shown in the right part of Figure 5d. Noteworthily, the GST rods deposited on top of the TiN electrode can be selectively accessed and modified by electric current pulses, as shown in Figure 5e.

### 4.2. Tunable/Switchable Lensing

Similarly to beam steering with tunable linear phase profile, switchable lensing can be achieved by producing a quadratic phase profile with moveable focusing. For schemes based on the P–B phase, the GST-metasurface hybrid varifocal lens can be constructed by translating the quadratic phase into spatially distributed nanorods or antenna with different orientations.

**Figure 6:**
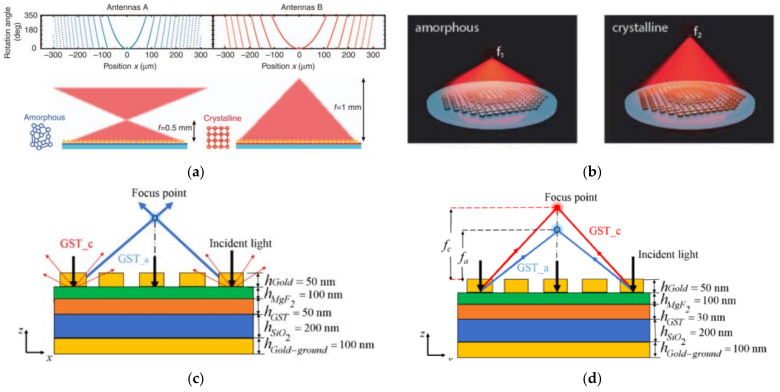
(**a**) The plasmonic metasurface composed of two sets of nanoantenna that interact with incident light alternately to produce a variable quadratic phase for bifocal lensing; (**b**) the artistic impression of a tunable metalens composed of GSST meta-atoms with bifocal length at 1.5 and 2.0 mm in the amorphous and crystalline states, respectively; (**c**) a type of mid-IR metalens with switchable focusing in the amorphous (focusing) and crystalline (defocusing) states and (**d**) tunable focal length in both states—(**a**,**b**) reproduced with permissions from [149,154]; (**c**,**d**) reproduced with permissions from [30].

As was also demonstrated by Yin et al. [49], shown in Figure 6a, a one-dimensional (1D) cylindrical bifocal metalens with a switchable focus at 0.5 mm and 1 mm was achieved in the amorphous and crystalline states, with antenna sets A and B being alternately dominant. Somehow, the resonant nature of sparsely distributed antenna gave rise to a low efficiency less than 10%. For improved efficiency, Shalaginov et al. proposed one all-dielectric varifocal metasurface lens based on Ge_2_Sb_2_Se_4_Te (GSST) Huygens meta-atoms on top of CaF2 substrate [154], as shown in Figure 6b. The phase error was minimized, and the optical efficiency was maximized in a combination of 16 discrete metatoms for quadratic phase sampling. At the incidence of mid-IR wavelength of 5.2 μm, the GSST atoms metasurface focus on varied focal lengths (1.5 and 2.0 mm) in the amorphous and crystalline states with the efficiencies above 20%.

Furthermore, a reflective metalens with switchable and tunable focusing with improved cross-polarization efficiency (up to 80%) was also demonstrated by Zhou et al. in a G2S2T5-integrated MIM architecture [30]. Working in the switchable mode, the device focuses with maximally cross-polarized reflectance (CPR) in the amorphous state and defocus with suppressed CPR in the crystalline state. While working in the varifocal mode, the GST–MIM metalens was optimized with moderately high CPR for variable focusing on both states. Bai and Yang et al. also demonstrated a tunable metalens with similar behaviors of duplex focusing in the near or middle IR range using the GST array of nanocuboids [155,156].

### 4.3. Tunable SAM–OAM Coupling

Revealed by Marrucci’s pioneering work [157], the spin angular momentum (SAM) of circular polarized wave/light can be converted into orbit angular momentum (OAM) in both optically inhomogeneous and anisotropic media. Therefore, the spin and orbit properties as well as spin–orbit couplings or interactions, i.e., SAM–OAM coupling or SOI for short, become additional degrees of freedom for the spatially structured and inhomogeneous optical field. As one of the basic optical process, SOI plays a key role in diverse SOI-based phenomena and applications [158,159], e.g., the spin-hall effect [160,161], P–B phase [161,162], spin-independent helical phase [163] and so on.

**Figure 7:**
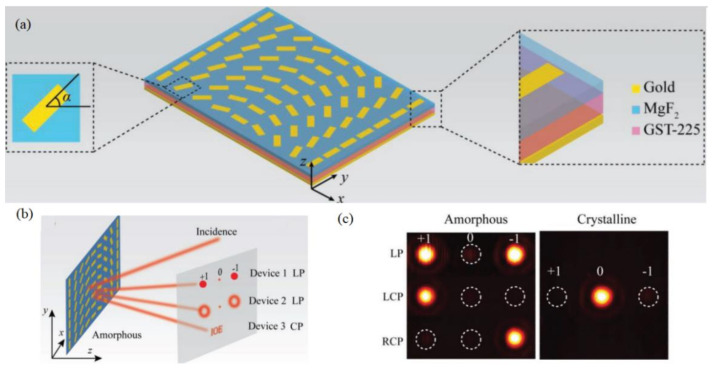
(**a**) Active metasurfaces with GST embedded in MIM setup for switchable SOI; (**b**) schematic of switchable SOI with the maximized cross-polarization in the amorphous state and (**c**) one device was demonstrated with switchable and selectable beam deflections. Left- and right-handed circular polarization (LCP and RCP, respectively) generated one beam with opposite deflections and linear polarization (LP) produced two symmetric deflections—(**a**–**c**) all figures are reproduced with permissions from [150].

In this trend, people in Luo’s group reported a diversity of SOI-based metadevices for different scenarios, e.g., achromatic SOI generation [162], achromatic and asymmetric wavefront shaping [164,165], Bessel beam generation [166], extraordinary Yang’s interference [167], meta-holography [22] etc. As for tunable or switchable SOI devices, a few phase change metadevices were demonstrated with dynamic SOI and the active tuning of the geometric phase profile [76,150]. In their first proposal, a type of MIM-G_2_S_2_T_5_ hybrid metasurfaces were demonstrated with switchable SOI, enabling three kinds of phase tailoring, i.e., the spin-hall effect, vortex beam generations and holography [150]. In the amorphous state, the required phase difference between two reflective components along two orthogonal axes of nanoantenna led to a maximized efficiency of cross-polarization, so that the anomalous reflections were observed with varied tailoring of the P–B phase, as seen from Figure 7c for beam deflection with a linear phase profile. In the crystalline state, the cross-polarization efficiency was minimized and the geometric phase or SOI-enabled phenomena disappeared or “switched off”.

**Figure 8:**
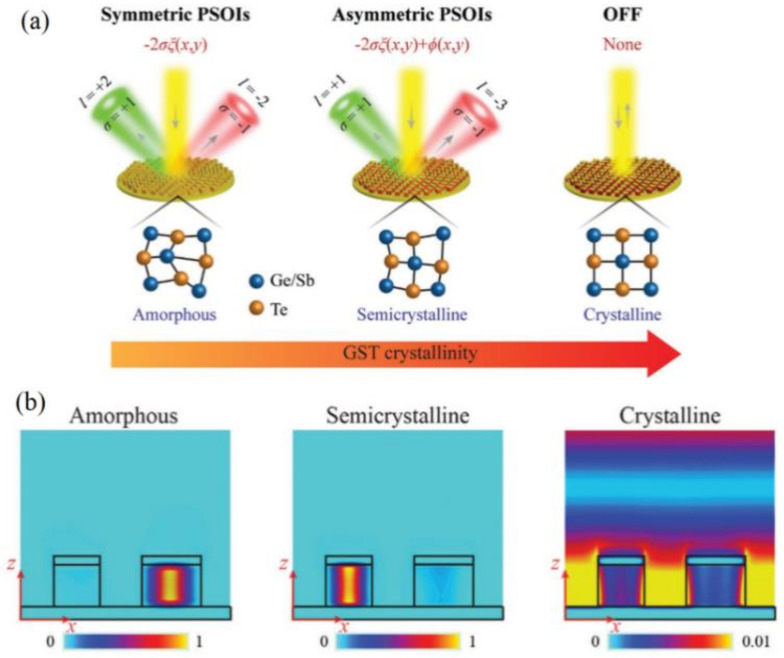
The multistate switching of SOIs by G_2_S_2_T_5_-hybrid metasurfaces composed of MIM nano-cavities: (**a**) three states with symmetric, asymmetric and “off” PSOIs; (**b**) the diatomic resonator used to confine the incident energy for the alternate tuning of the propagation phase—all figures in (**a**,**b**) are reproduced with permissions from [76].

Very recently, the same group further investigated the multistate switching of photonic spin–orbit interactions (PSOIs) by proposing another type of G_2_S_2_T_5_-integrated metasurface composed of MIM nano-cavities [76]. As shown in Figure 8a, by the phase transition of G_2_S_2_T_5_ embedded as the middle spacer in the MIM setup, the propagation phase can be actively tuned in combination with the fixed P–B phase for overall phase tailoring as well as spin control. By tuning the crystallization levels of G_2_S_2_T_5_, the original amorphous state with symmetric SOI can be switched into more intermediate states with opposite topological charges and asymmetric SOIs. Also shown in Figure 8b, the exemplary semicrystalline state can be created for asymmetric SOIs by using the diatomic nanofin resonators that alternately confined incident fields for the different tuning of the reflected propagation phase. Upon a phase transition to the crystalline state, the device can be totally “switched off” with only normal specular reflection.

### 4.4. Tunable Vortex Beam and Holography

As a special subcategory of phase control, helical phase and even arbitrary phase generation are widely embraced in optical vortex and holography. By hybridizing PCMs into metadevices with predefined P–B phase controls, helical and holographic phase control with active tenability can be demonstrated straightforwardly.

**Figure 9:**
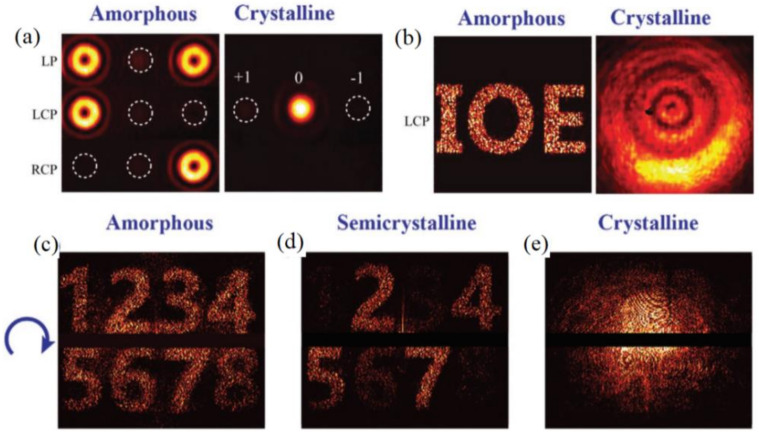
(**a**) Tunable helical phase and (**b**) holography for metadevices with switchable SOIs; the tunable holography achieved by the devices with multistate switching SOIs in the (**c**) amorphous, (**d**) semicrystalline and (**e**) crystalline states, respectively—all figures in (**a**–**e**) are reproduced with permissions from [150].

As also reported by Luo’s group [150], switchable vortex beams and meta-hologram were also demonstrated by the mechanism of switchable SOIs discussed in Section 4.3. In the amorphous state, the incidence of LCP or RCP led to an anomalously reflected vortex beam with reverse deflections, but linearly polarized (LP) incidence gave rise to two beams with helical phase simultaneously (shown in Figure 9a). A P–B phase tailoring also made a hologram of the characters “IOE” displayed in the far field, shown in Figure 9b. In the crystalline state, both helical and holographic phases were “switched off” with only normal specular reflection, shown in the right parts of Figure 9a,b.

Very recently, Yuan’s group also presented one type of dielectric metasurface with dynamic wavefront tunability for optical vortex and holography by using GSST nanopillars for metamolecule design [90]. Phase modulation is enabled in the amorphous state until it covers nearly the entire 2π range by periodically arranged nanopillars with varying diameters. Upon a GSST phase transition to the crystalline state, the phase modulation was disabled. Furthermore, the multilevel modulation of phase profile was achieved by selectively controlling the phase transition of each bi-state GSST nanopillar in the molecule composed of four GSST pillars with fixed diameters.

## 5. Continuous and Quasi-Continuous Metasurfaces

For the sake of active photonic applications where in situ electrical control is highly desirable, e.g., integrated optoelectronics, continuous meta-atoms that simultaneously act as electrodes for the local access of electrical pulses are more viable for practical use. Towards this trend, myriads of continuous or quasi-continuous meta-atoms were employed to construct the PCM hybridized active meta-devices, e.g., grids or nanoholes [113,114] for tunable extraordinary transmission (EOT), gratings [29,36], trapezoids [168,169], and catenaries [162,167].

### 5.1. Gratings

For the most common structures, gratings or nanoslits that are one-dimensional (1D) and continuous were intensively employed in the MIM-based metasurfaces for tunable absorption by Carrillo et al. [170,171], tunable reflection using the ITO-embedded setup [10], and GSTs-induced IR beam steering [29] etc.

**Figure 10:**
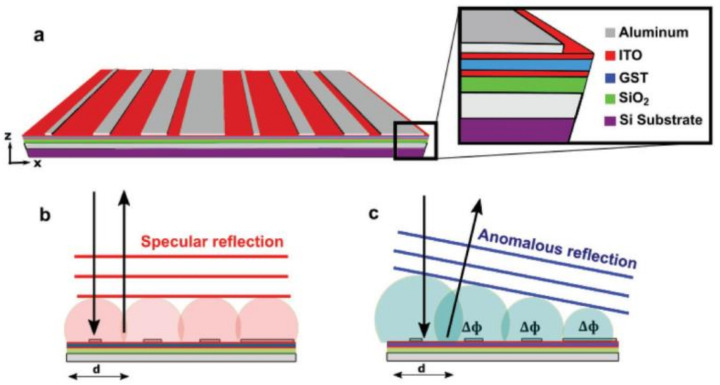
(**a**) Continuous metasurface with a 1D array of antennas configured in the MIM setup for beam steering by Wright’s group; (**b**) normal incidence and specular reflection in the crystalline state and (**c**) anomalous reflection in the amorphous state—all figures in (**a**–**c**) are reproduced with permission from [29].

**Figure 11:**
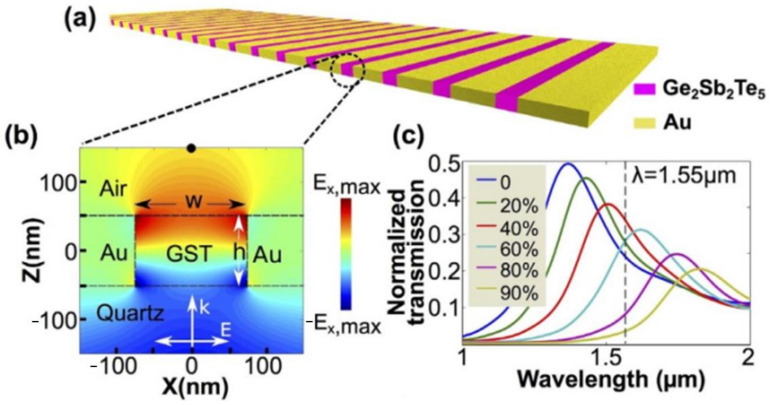
(**a**) Continuous metasurface with a 1D array of nanoslits filled with G_2_S_2_T_5_; (**b**) the schematic of F–P mode between the Au nanoslit intervals; (**c**) a varied crystallization level leading to a series of evolved transmission spectrum, enabling the multistate tuning of focusing—all figures are produced with permissions from [172].

As a typical proposal from Wright’s group [29], a 1D array of antennas or zone plate was used as the top layer in the MIM setup for beam steering, shown in Figure 10a. Anomalous reflection towards a predefined angle was achieved in the amorphous state by locally arranging 1D antenna with varied widths to generate a linear phase gradient. A phase transition to the crystalline state driven by the 405 nm laser pulse heating steered the beam reflection towards the normal angle of specular reflection, shown in Figure 10b. In a similar example, shown in Figure 11a, Chen et al. demonstrated a G_2_S_2_T_5_ hybrid varifocal metalens with tunable focusing by embedding G_2_S_2_T_5_ into the intervals of nanoslits for phase change-based state switching [172]. The varied crystallization level led to different transmissions and phase modulations for the active tuning of focal lengths.

Specifically, except for most work demonstrated in the IR range, Behrad et al. investigated the GST phase transition-induced dynamic plasmonic resonances in the UV and high-energy visible range (UV–HEV) [117,118]. A layered composite grating of G_2_S_2_T_5_ sandwiched between the two protective layers of ZnS/SiO_2_ was constructed to exhibit tunable reflection resonances with quality factors up to Q ~ 15 due to the transparency (low losses) of ZnS/SiO_2_ [118].

Moreover, one type of continuous metasurface constructed with sinusoidal nanostrips was demonstrated for SOI-based phase modulation in scattering engineering by Guo et al. [173]. For high-quality OAM generation or spin–orbit interaction as discussed above, a quasi-continuous metasurface integrated with circular gratings and discrete scatter was also proposed in the GHz range [174].

### 5.2. Catenary Structures

As one type of architectural structure with specified mathematical and mechanical form by Robert Hooke in the 1670s, a catenary was first introduced into optics in 2015 by Pu et al. [162]. As a quasi-continuous structure, the optical catenary was adopted as one type of special meta-atom to construct a diversity of metasurfaces with high-efficiency phase tailoring [18,166,175,176,177,178,179].

**Figure 12:**
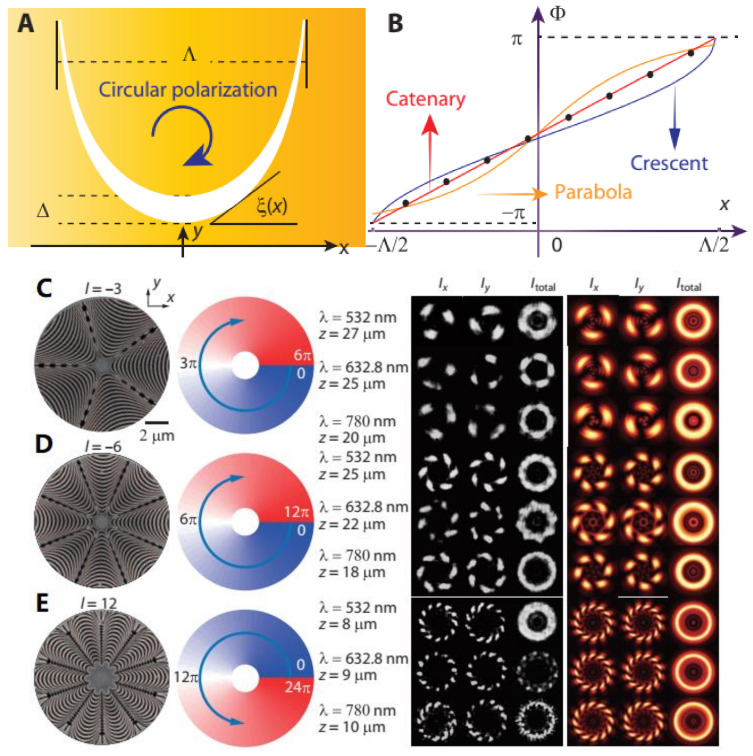
Catenary structure as a special type of meta-atom for P–B phase modulation under the incidence of circular polarization; (**A**) the catenary atoms with varying tangent angle from −π/2 to π/2 and (**B**) corresponding phase tailoring from −π to π, and topological charges of (**C**) −3, (**D**) −6 and (**E**) 12 by the catenary-based OAM generator (first column), in accordance with the helical phase profile (second column) and the simulated (third column) and experimentally demonstrated intensity pattern. All figures are reproduced with permission from [162].

For the pioneering work by Pu et al. [162], a single catenary meta-molecule with varying tangent angle from −π/2 to π/2 can achieve the full phase modulation from −π to π, shown in Figure 12b. Therefore, by the predetermined arrangement of the array of catenary atoms, the phase distribution along the predetermined direction can be tailored in specified manner. For example, a helical phase for vortex beams can be produced by arranging the catenary atoms in varied columns along a circular loop with the perfect axial symmetry, shown in Figure 12c–e. Obviously, as can be seen from the second column of Figure 12, varied cycles or columns of catenary for a circle enable phase profiles with different gradients or “density” for OAMs with different topological charges. Furthermore, catenary atoms were also used to construct one type of meta-axicon for high order Bessel beam generation with highly focused OAM propagation. Subsequently, Li et al. specified this process in detail by optimally configuring catenary atoms in different arrangement to produce Bessel beams with varied orders and a helical phase profile with varied topological charges [166].

Following that trend, myriad catenary based meta-devices were demonstrated, such as a deflector or director [177], lensing [178] and polarizer [179]. In addition, the resonant optical field in between neighboring meta-atoms or resonators was also found to follow the rule of catenary function, which revealed a new avenue for dispersion engineering [13], SOI [180] and perfect absorber [176] etc.

### 5.3. Grids or Fishnets

The orthogonal or non-orthogonal grids or fishnet structures with square or circular nanoholes can be regarded as the 2D counterpart of gratings or nanoslits that are frequently used for diverse metamaterials and metadevices. Among those continuous metasurfaces, the ones constructed by grids intrinsically exhibit complementary responses with respect to those constructed by the squares, crosses, or circular pillars.

Since its emergence decades ago, the fishnet structures or metallic grids had been intensively used in metal mesh filters [181,182], frequency selective surfaces [182,183] and the negative index metamaterials [184,185] in early days. In recent years, metasurfaces based on 2D grids or fishnet were demonstrated for optical activity or circular dichroism [140,186], tunable EOT [114,140] and dispersion engineering [185], etc.

**Figure 13:**
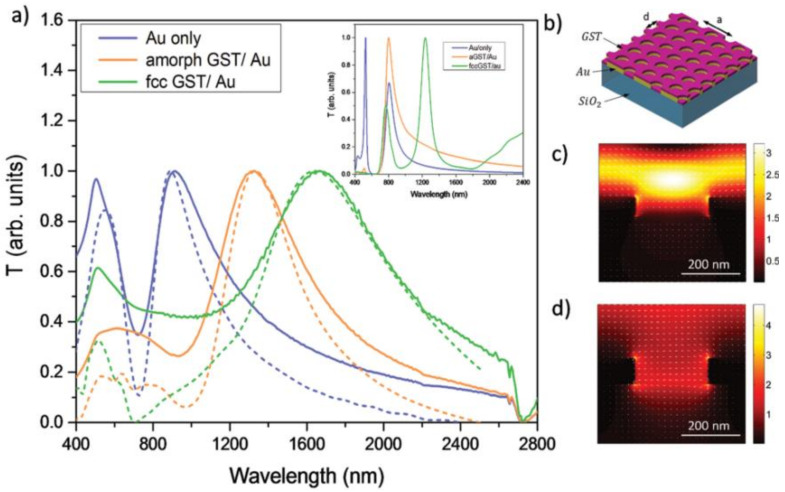
The GST-integrated fishnet metasurfaces constructed by a circular holes array for actively tunable EOT [114]; (**a**) transmission spectra with moveable peaks of EOT for plasmonic resonance by bare metallic hole and the other two with amorphous and crystalline GST on top; (**b**) the device setup; (**c**,**d**) the field distribution for (**c**) off-resonance and (**d**) on-resonance mode—all figures are reproduced with permission from [114].

Recently, the PCMs-integrated grids were employed by Rude et al. [114], as a typical example for an active metasurface with the broadband tuning of EOT in the visible and near-IR range. In contrast to the MIM setup, GST grids were used on top of metallic grids with silicon dioxide underneath as the substrate, shown in Figure 13b. Upon a phase transition from the initial amorphous state to the crystalline state, which can be triggered by optical excitation (35 fs laser pulse at 800 nm, fluence of 5 mJ/cm ^2^) or electrical stimuli (DC current for 20 s at 3.5V, 1.5 A), the samples demonstrated movable EOT peaks of plasmonic resonance.

Noteworthy, continuous metasurfaces using grids structures were also demonstrated in THz range with the connected metallic meta-atoms that facilitate electrical access for phase change control [99,187]. Typically proposed by Zhou et al. [99], the grids pattern was constructed by interleaving one array of interdigital metal slits with another orthogonal array of VO_2_ slits. The metallic grids act as both the resonators and electrodes for phase change of VO_2_ slits. In another VO_2_-grids hybrid framework by Cai et al. [187], the meta-atoms of split ring resonators (SRRs) were interconnected by a conductive wire that strings all SRRs at one side. All VO_2_–filled SRRs were electrically excited and the overall transmission spectra were actively tuned by different temperatures under Joule heating. The inherent hysteresis behavior of VO_2_ enables a multistate amplitude modulation, potentially in favor of applications as the electrically controlled digital photonic devices, i.e., the digital optoelectronic integration.

### 5.4. Others

Other than the structures discussed above, a few other metamolecules of antennas or slits with diversely structured geometries were also frequently used to construct the continuous or quasi-continuous metasurfaces, e.g., trapezoids [168,169,188], crescents [189,190], and zigzags [191,192,193], just to name a few.

**Figure 14:**
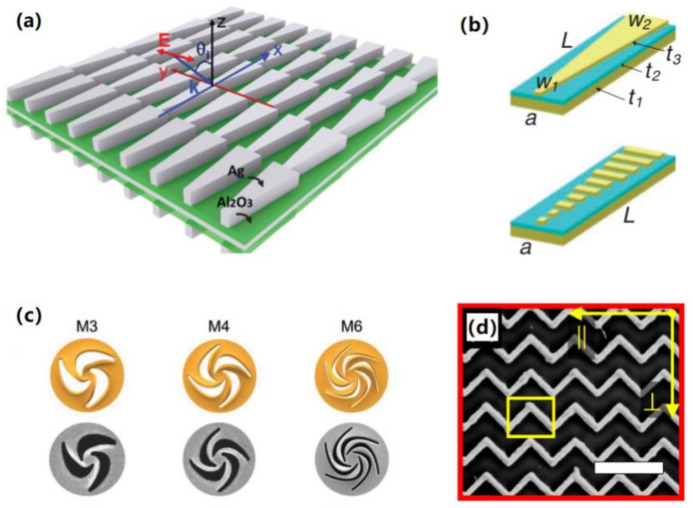
Typical examples of metasurfaces constructed by continuous or quasi-continuous meta-molecules, such as (**a**) trapezoids (**b**) that be regarded as combined rods with varied widths, (**c**) crescents and (**d**) zigzags array—(**a**–**d**) are reproduced with permissions from references [169,188] (Copyright (2014) The Japanese Society of Applied Physics), [190,193] sequentially.

For one typical candidate of metamolecules with the structural characteristic of spatial continuity, the trapezoid antennas intrinsically offer distortion-free and continuous phase tailoring for the incidence of linearly polarized waves. As an example, Qiu’s group demonstrated one type of continuous metasurface for high-efficiency anomalous wave bending [169], to conquer the efficiency issue existing for most traditional metadevices, shown in Figure 14a. By aligning the parallel edges of trapezoids along the polarization direction of LP incidence, a broad band phase modulation covering almost the entire visible range can be obtained with combined resonances of different cross-sections at varied wavelengths. The spatial continuity in phase modulation was further specified by regarding each single trapezoid antenna as a series of rods with continuously varied widths [188], shown in Figure 14b. For coherent control in multiple anomalous scattering, the trapezoid-shaped slit metasurface was also numerically studied [168].

Similarly, the quasi-continuous metasurface composed of crescent antenna was typically used for circular dichroism (CD) control using the MIM setup [189]. As shown in Figure 14c, Li’s group also presented one type of metasurface with spirally combined crescent-shaped meta-atoms for versatile Fano-based CD control [190]. However, as revealed earlier in 2015 by Pu et al. [162], such quasi-continuous atoms as crescent or parabolas caused nonlinear phase tailoring as compared to catenary meta-atoms.

Furthermore, as proposed by Buchnev et al. from Zheludev’s group [191,192], another type of continuous atoms, the zigzag atoms of nanowires, were actually used as connected “V”-shaped antenna array enabling electrical access as electrodes, such as the liquid crystal hybridized metamaterials or devices with active electro–optical control. As shown in Figure 14d, the zigzag nanoslits or nanowires were also used to construct the metasurface that is immune to high-order diffraction for coherence recognition [193]. In contrast to the spectra caused by coherent waves, incoherent illuminations caused a local resonance or split to global resonant band that was exclusive to the continuous atoms.

## 6. Conclusions and an Outlook

In summary, we briefly reviewed the PCMs-integrated active metasurfaces for dynamically tunable phase and amplitude regulation, especially featuring those with continuous or quasi-continuous meta-atoms enabling convenient electrical control as well as the compact electro–optical or optoelectronic integration. Basically, two types of PCMs are currently being used as the mainstream candidates for active photonic devices from the visible to THz range. In contrast to VO_2_ that is volatile in the active control, the chalcogenide PCMs of GST alloy exhibited great potential and intensive applications for the non-volatile and active control of diverse photonic devices, due to their advantages in large cycles of reversible phase change, long-term stability and distinct optical and electrical contrast in the amorphous and crystalline states. As discussed above, GST alloys have been immensely used in diverse hybridized architectures or directly in dielectric metasurfaces for dynamic tuning of amplitude control (e.g., tunable absorption, reflection, transmission, thermal emission and circular di-chroism etc.) and phase tailoring (e.g., tunable deflection, lensing, vortex beam and holography etc.). However, the PCMs-based active metasurfaces just started to set sail and diverse architectures of device with versatile functionalities are still emerging. In this situation, a few foreseeable challenges or directions may come out to be addressed toward multifunctionality or versatility, long-term reliability and ultra-compact integration for future trends.

First, it is imperative to further expand the passive functionalities of current metasurfaces for different purposes or figures of merits, e.g., efficiency, broadband control, phase continuity and even versatile smart controls including the functional multiplexing of wavelength, polarization and resonant modes etc. Moreover, it is highly desirable to endow such passive versatility with more degrees of freedom in the dynamic control of amplitude, wavefront or polarization. Second, multi-state active control achieved by the multi-level crystallization of PCMs enables a few intriguing phenomena and emerging metadevices. So, some GSTs or VO_2_-based applications may evoke further investigations into the phase change process with more intermediate states between the common bi-state controls. A few examples can be found in previous discussions [76,187,194]. Third, although the commonly used GST alloys exhibit the advantageous contrast of optical constants, relatively high optical loss still exists for most of them, especially in the visible and near-IR range. Therefore, GSTs with exquisitely optimized compositions and thus minimized optical losses would attract more research of interest in this field. For an example, a new class of PCMs, namely Ge–Sb–Se–Te (GSST) [90,91], was recently found to exhibit well transparency and low loss in an extremely broadband range (1–18.5 µm) by adequately sacrificing the switching speed, and somehow shows prospects for the emerging infrared photonic devices.

Finally, on-chip photonic and electronic integration is becoming an inevitable trend, especially for ultra-compact metadevices with natural CMOS-compatibility for monolithic integration. Specifically, given the actively reconfigurable functionalities by thermal, optical and electrical stimuli, it is somewhat highly desirable to practically achieve fully reversible and real-timely active control by the in-site photonic or electrical stimuli, especially the electrical control that facilitates monolithic electro–optical integrations. Therefore, metasurfaces with continuous atoms for electrical connectivity are preferred for future device integration. Furthermore, for certain applications of integrated photonic devices with more active versatility, it is highly urgent to introduce the locally selective access of electrical stimuli for addressable phase transition of individual atoms, especially for future monolithic on-chip electro–optical integration.

## Data Availability

Data sharing not applicable.
